# Perivitelline Membrane‐Bound Sperm as a Source of Paternal Genomic DNA to Inform Breeding Male Marine Turtle Genetics and Demographics

**DOI:** 10.1002/ece3.73115

**Published:** 2026-02-15

**Authors:** Brian M. Shamblin, Cheryl L. Sanchez, Sean M. Perry, Simona A. Ceriani

**Affiliations:** ^1^ Warnell School of Forestry and Natural Resources University of Georgia Athens Georgia USA; ^2^ Florida Fish and Wildlife Research Institute Florida Fish and Wildlife Conservation Commission St. Petersburg Florida USA; ^3^ Mississippi Aquarium Gulfport Mississippi USA; ^4^ College of Veterinary Medicine Midwestern University Glendale Arizona USA

**Keywords:** breeding sex ratio, chelonian, mating system, multiple paternity, sea turtle

## Abstract

Sex in marine turtles is determined by incubation conditions, raising concerns of population feminization and loss of genetic diversity due to warming temperatures. Demographic data on breeding males are limited due to their relative inaccessibility. Hatchling sampling can inform multiple paternity (MP) and breeding sex ratios (BSR) but is logistically intensive, limiting the number of nests and populations analyzed. Here, we present a novel approach to characterize successfully breeding males by genotyping sperm trapped in the perivitelline membrane (PVM) surrounding the yolk of a single egg per clutch. We compared maternal genotypes via eggshells with PVM extract genotypes from 27 loggerhead turtle ( *Caretta caretta* ) eggs and 13 green turtle ( *Chelonia mydas* ) eggs from Melbourne Beach, Florida, USA, at 16 and 13 microsatellite loci, respectively. Sampled offspring genotypes (620 loggerhead and 1117 green turtle) provided ground‐truthing of PVM sperm allele detections. Paternity analyses resolved 38 loggerhead and 29 green turtle sires, all but two of which were correctly inferred via PVM allele calls. All paternal alleles representing single paternity clutches and primary contributors of multiply sired clutches were detected in the PVM genotypes except in one case of maternal DNA swamping of the PVM. Seven of eight sires that contributed ≤ 11% of offspring were detected via PVM genotyping, comparable to inferences based on sampling 20 hatchlings per nest. PVM‐inferred paternal genotypes from single‐paternity clutches were > 99% concordant with reconstructed sire genotypes in both species. Although destructive to a single egg per clutch, this method is non‐invasive to nesting females and hatchlings. Its scalability across space and time enables long‐term monitoring of MP and BSR, key demographic metrics for assessing population viability under climate change. Male genotypes generated from the PVM in single paternity nests may also inform population assignment, assessments of genetic connectivity, and relatedness analyses.

## Graphical abstract

Breeding male marine turtles are poorly characterized due to their inaccessibility. Here, we demonstrate that genotyping sperm trapped in the perivitelline membrane of a single egg permits assessment of multiple paternity and direct paternal genotyping in singly sired clutches. This approach is scalable spatially and temporally and should facilitate estimation of breeding sex ratios and male demographic parameters.

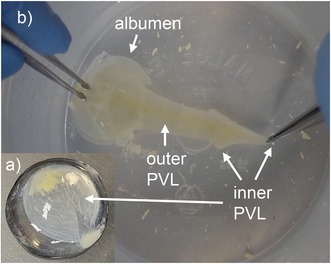

## 1 Introduction

Rapid global climate change may pose a threat to species with environmental sex determination, like chelonians (Butler 2019; Lockley and Eizaguirre 2021). All marine turtles display an environmental sex determination pattern in which cooler, wetter nests produce more males and hotter, drier nests produce more females (Wibbels 2003; Laloë et al. 2023). Rising incubation temperatures may pose a threat to fertility and genetic diversity via breeding male constraints. Long‐term feminization trends have been documented in Florida (USA), a globally important rookery for three species of marine turtles (Ceriani and Casale 2025). Genetic and endocrine data from Bermuda foraging grounds suggest that the juvenile green turtle ( *Chelonia mydas* ) aggregation sex ratio is becoming increasingly more female‐biased (Meylan et al. 2024). A similar genetic and endocrine approach applied to green turtles foraging along the Great Barrier Reef in Australia suggested that essentially only female offspring have been produced for the past two decades at Raine Island, one of the largest rookeries in the world (Jensen et al. 2018). Previous mitochondrial DNA analyses of breeding males and females in eastern Australia demonstrated strong natal philopatry to courtship sites adjacent to their respective natal beaches in both sexes (FitzSimmons et al. 1997), thus raising concerns of male limitations as the current breeding males senesce. These scenarios evoke broader questions about marine turtle mating systems. What is the minimum number of males necessary to maintain genetic diversity and ensure fertilization? How does the scale and magnitude of male natal homing versus straying vary among species and populations? These questions underscore the need for better data on breeding male demographics and spatial ecology (Maurer et al. 2021; Hays et al. 2022).

Identification of paternal alleles via live hatchling sampling has been utilized to identify multiple paternity (MP) within clutches and estimate breeding sex ratios (BSR) in marine turtles. This approach is logistically intensive as it requires restraining caging and regular nocturnal monitoring of nests in anticipation of hatchling emergence. Although efforts are made to minimize stress on hatchlings, the effects of handling and sampling on hatchling survival are unknown. A standard methodology is attempted sampling of 20 hatchlings per nest (e.g., Moore and Ball 2002; Howe et al. 2018) but often nests with fewer offspring are included in calculations without including the associated metadata, making it difficult to directly compare studies. The logistically intensive nature of this sampling effort has limited these studies to relatively few nesting sites across species globally (Lee et al. 2018). Loggerhead turtles ( *Caretta caretta* ) in the Northwest Atlantic are among the best represented with four nesting sites characterized (Moore and Ball 2002; Lasala et al. 2013, 2018; Silver‐Gorges et al. 2024). These studies have yielded MP estimates from as low as 17% with 1.3 males per clutch in northwestern Florida in 2022 (31 nests, Silver‐Gorges et al. 2024) to as high as 75% and 2.7 males per clutch in Georgia during 2008–2010 (72 nests, Lasala et al. 2013). Because none of this sampling was conducted contemporaneously, spatial and temporal variation in inferred breeding sex ratios are confounded. Moreover, the lack of spatial and temporal overlap precludes inferences of broader scale genetic connectivity. Nuclear gene flow can occur via male‐mediated gene flow, where males stray to non‐natal courtship areas, as well as via migration‐mediated gene flow, where mating of turtles from different populations occurs along migratory routes, even in the presence of natal philopatry of both sexes (Karl and Bowen 2007).

A more efficient technique for characterizing breeding males is needed for broader implementation of male demographic and genetic studies to inform conservation actions. Genotyping of sperm trapped in the perivitelline membrane (PVM) surrounding the yolk was presented as a method to identify sires to study sperm competition in birds (Carter et al. 2000). Two expected concerns with this approach are swamping of sperm DNA by maternal or embryonic DNA during PCR and preferential amplification of shorter microsatellite alleles (Carter et al. 2000). A subsequent controlled experiment in artificially inseminated chickens substantiated this concern, with larger alleles failing to amplify when a secondary male's semen comprised only 20% of the sperm mixture (Martínez and Burke 2003). Sperm allele dropout was common in general, with complete paternal genotypes recovered from only 41% of PVM samples. Despite removing the embryo prior to DNA extraction, maternal DNA was detected in the majority (86%) of chicken PVMs, suggesting maternal leakage from a source other than the embryo and associated tissues (Martínez and Burke 2003).

In chelonian systems, characterizing PVM‐bound sperm has received attention as a tool for assessing fertility in undeveloped eggs. Croyle et al. (2016) demonstrated detection of PVM‐bound sperm via microscopy as a means of differentiating infertility from early embryonic death in eggs that failed to exhibit development. Amplification of a mitochondrial DNA fragment from PVM‐extracted DNA suggested the presence of paternal DNA (Croyle et al. 2016), but this was not confirmed via maternal and paternal nuclear DNA comparisons. PVM‐bound sperm detection has been explored for confirming fertility in marine turtle eggs that fail to develop (Phillott and Godfrey 2020; Turla and Wyneken 2024). Here we assessed whether genotyping of PVM‐bound sperm could be a robust technique for inferring paternal contributions in marine turtle clutches. We maximized offspring sampling depth where possible to provide a high‐resolution view of paternal contributions in multiply sired clutches. As far as we are aware, this was the first attempt to genotype paternal nuclear DNA from PVM‐bound sperm in a chelonian. We explored two primary questions that affect the utility of this technique for informing BSR and genetic characterization of breeding males: (1) Can sperm alleles detected in the PVM of a single egg be used to estimate the true number of sires in multiply sired clutches? and (2) Can robust paternal genotypes be directly inferred from the PVM in singly sired clutches?

## 2 Methods

### 2.1 Field Sampling

Loggerhead turtle and green turtle nests were marked along a 2‐km stretch of beach (between 28.00866, −80.52847 and 27.99272, −80.52031) on the Archie Carr National Wildlife Refuge (ACNWR) in Melbourne Beach, Florida. We collected 27 loggerhead turtle eggs and 13 green turtle eggs (one egg per clutch) the morning following oviposition and stored each in a small plastic bag (Table S1). Eggs were refrigerated at 4°C for 0–5 days prior to PVM isolation.

To support PVM inference calls and assess relative male contributions, we sampled live hatchings and salvaged dead hatchling and embryo tissue (see Section 2.3). Once marked nests reached 45 days of incubation, we outfitted them with restraining cages. The cages were deployed just before sunset each evening and were checked three times throughout the night. Cages were removed each morning around sunrise. All hatchlings were removed if found and were either sampled on the beach next to the restraining cage, weather permitting, or were taken to the lab (2‐km maximum distance) and sampled. We deployed cages on each nest until an emergence was observed, and cages were deployed the two nights following the first emergence to catch any subsequent emergences to maximize sampling opportunities. We sampled the rear margin of the front flipper using a 1.5‐mm biopsy punch. Samples were stored in 95% non‐denatured ethanol until DNA extraction. All hatchlings were released in groups when possible. All methods followed Florida Fish and Wildlife Conservation Commission guidelines (FWC 2016).

We evaluated all marked nests 3 days after the first hatchling emergence (regardless of whether any live hatchlings were collected). Any dead hatchlings without significant decomposition and whole, unhatched eggs were taken to the lab for sampling. Once in the lab, dead hatchlings were skin sampled with a 1.5‐mm biopsy punch. We opened all unhatched eggs. Embryos with enough flipper to biopsy (stages 25–31, Miller et al. 2017) were sampled with a 1.5‐mm biopsy punch, and less developed embryos (stage < 25) were stored whole in 1.5‐mL cryovials. Offspring with obvious signs of significant decomposition were not sampled. All tissue samples were stored in 95% non‐denatured ethanol prior to DNA extraction.

### 2.2 Laboratory Methods

#### 2.2.1 PVM Isolation

**FIGURE 1:**
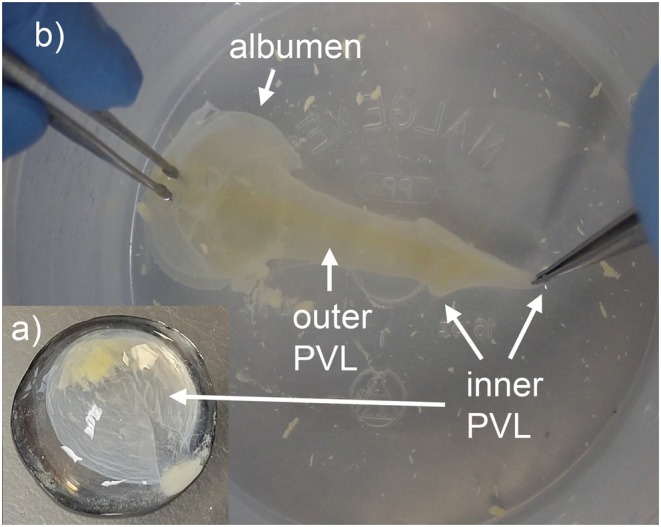
Remaining constituents of a loggerhead turtle egg following shell, embryo, and yolk removal. The inner perivitelline layer (PVL) is opaquer and more rugose than the remaining egg components, resembling a contact lens (a). When the perivitelline membrane is stretched, the inner PVL forms a slight “arrowhead” shape that is distinguishable from the outer PVL that forms the “shaft” of the arrow (b).

Eggs were rinsed to remove all sand. Attempts to remove the embryo followed methods of Gárriz et al. (2020). Following embryo removal or when no embryo could be identified, most of the yolk was then scooped out of the PVM. The remaining albumen and PVM were transferred to a container, and the residual yolk was rinsed from the PVM using tap water (similar to Turla and Wyneken 2024). The albumen and PVM were transferred to a glass petri dish with a small amount of water. We isolated the inner layer of the PVM from the outer layer and albumen by grasping it with forceps and stretching it. When stretched, the inner PVM layer, which previously surrounded the yolk, formed a partially opaque, often slightly yellow “arrowhead” that was consistently less transparent and more rugose than the outer perivitelline layer (the “arrow shaft”) and albumen (Figure 1). We attempted to trim away any remaining gelatinous albumen or outer PVL that adhered to the inner PVM layer. We cut the trimmed inner PVM layer using forceps and sharp scissors and stored them in 2‐mL microcentrifuge tubes as two to three aliquots of approximately 250–350 μL each. These were frozen at −20°C prior to DNA extraction. Eggshells were stored in 95% non‐denatured ethanol at room temperature.

#### 2.2.2 DNA Extraction

Maternal DNA was isolated from eggshells using a modified Qiagen DNEasy blood and tissue protocol as previously described (Shamblin et al. 2011). PVM extractions were conducted in a similar manner by digesting a PVM aliquot in 300 μL of tissue lysis buffer, 20 μL of proteinase K, and 20 μL of 10 mM dithiothreitol (DTT). PVM DNA was eluted in 80 μL of elution buffer. For early‐stage embryos, the entire embryo was digested using Qiagen DNEasy blood and tissue kits per the eggshell extraction protocol.

For hatchling and late‐stage embryo biopsy samples, ethanol was evaporated from PCR plates containing skin biopsies. Plates were treated with 50 μL of 20% Chelex‐100 resin and heated to 99.9°C on a thermal cycler for 20 min. Following thermal cycling, the supernatant was passed over filter plates with 0.45 μM pore size using a multichannel pipettor to elute DNA and capture chelex resin and any tissue debris. Eluted DNA was diluted 1:1 with water prior to PCR.

#### 2.2.3 Genotyping

Loggerhead turtle samples were genotyped at 16 microsatellite loci in three multiplex reactions (Table S2) as previously described (Shamblin, Dodd, et al. 2021). These loci were chosen as they have been employed in a long‐term genetic tagging effort for Northwest Atlantic loggerhead turtles (Shamblin et al. 2017, Shamblin, Dodd, et al. 2021). Green turtle samples were genotyped at 13 microsatellite loci in three multiplex reactions that were combined into two fragment analysis plates (Table S2). These loci were chosen because they had been previously demonstrated to be highly polymorphic in western Atlantic green turtles (Shamblin, Berry, et al. 2012; Naro‐Maciel et al. 2014) and because several of the loggerhead loci were not informative in green turtles (Shamblin et al. 2009). Eggshell, late‐stage embryo, and hatchling PCRs were conducted in 10‐μL reactions as previously described (Shamblin, Dodd, et al. 2021). PVM and early‐stage embryo PCRs were conducted in 20‐μL reactions at the same final concentrations as 10‐μL reactions but with modified dilutions (Table S2). Where sufficient PVM was available, PVM samples with failed or weak amplification were subjected to a second round of DNA extraction and PCR amplification using a second PVM aliquot.

### 2.3 Data Analyses

We confirmed and added GeneMapper calls for PVM alleles by visually comparing them with maternal electropherograms from eggshells from respective nests. For comparative purposes, we recorded all PVM alleles, regardless of whether they were interpreted as paternal or maternal. For tetranucleotide loci, allele calling was relatively straightforward due to single stutter peaks that were typically < 5% of the RFU of true peaks. Some green turtle dinucleotide loci were more challenging to call due to more complex (e.g., stair‐step) stuttering patterns. For all loci, we used peak morphology from eggshell and offspring samples where a maximum of two alleles were expected per locus to calibrate baseline stuttering patterns for PVM calling.

**FIGURE 2:**
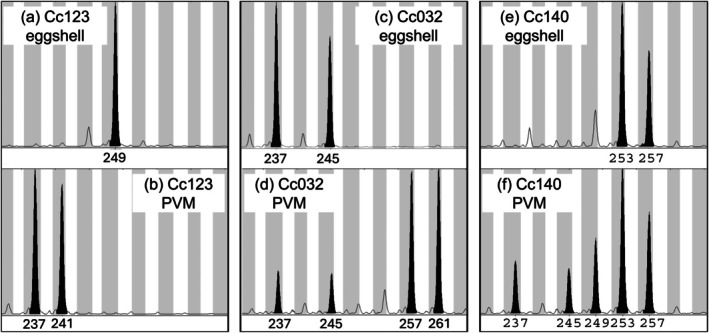
Microsatellite electropherograms for loggerhead turtle eggshell (upper panel) and perivitelline membrane (PVM) DNA (lower panel) pairs for three eggs at locus 74 (CcP7E11). Panels a, c, and e illustrate maternal genotypes generated from eggshell DNA from nests Cc123, Cc032, and Cc140, respectively. Panel b illustrates a strictly paternal PVM genotype for clutch Cc123 with no sign of maternal allele 249. Panel d illustrates an admixed PVM genotype with paternal alleles 257 and 261 with a stronger signal than maternal alleles 237 and 245. Panel f illustrates an admixed genotype with weaker paternal alleles 237, 245, and 249 relative to maternal alleles 253 and 257.

The first step of PVM interpretation was determination of the primary signal as paternal, admixed, or embryonic. Some PVMs were easily identifiable as strictly paternal because no maternal alleles were present across multiple loci (Figure 2a,b). Where PVM extracts yielded apparently admixed paternal and maternal alleles, we assessed their relative signal strengths by comparing PVM and eggshell genotype pairs across all loci (Figure 2c–f). Where admixed alleles were present, we attempted to determine if the contamination was maternal (e.g., both maternal alleles present for some loci) or embryonic (only one maternal allele ever present). Embryonic swamping of the PVM was assumed when only two alleles were present at most loci and when a maternal allele was present across all microsatellite loci examined.

**FIGURE 3:**
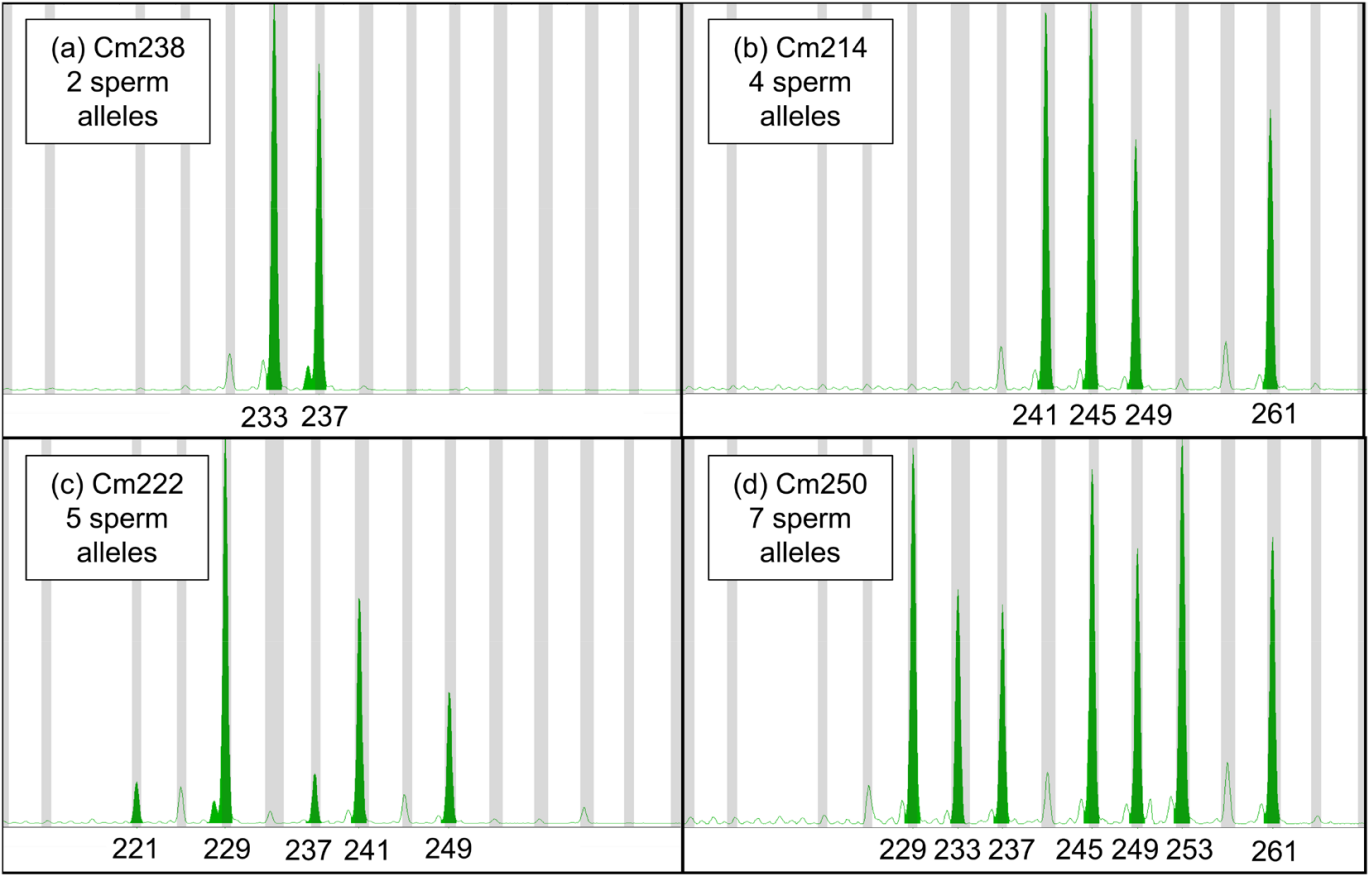
Microsatellite electropherograms for green turtle sperm alleles for four eggs at locus CHMY01. Panel a illustrates two paternal alleles in clutch Cm238, corresponding to a single sire. Panel b illustrates four paternal alleles in clutch Cm214, corresponding to two sires. Panel c illustrates five paternal alleles in clutch Cm222, corresponding to three sires. Panel d illustrates seven paternal alleles in clutch Cm250, corresponding to four sires.

We estimated the minimum number of sires of each clutch based on the number of non‐maternal PVM alleles across loci. Where PVMs were strictly paternal with no sign of maternal contamination, we considered all alleles as paternal. Where PVMs were admixed, we conservatively excluded all maternal alleles (given potential uncertainty in defining allele sharing versus maternal contamination) to minimize the risk of overestimating sires. If one or two non‐maternal alleles were present at all loci (Figure 3a), one sire was inferred. If three or four non‐maternal alleles were present at any loci (Figure 3b), two sires were inferred. If five or six paternal alleles were present at any loci (Figure 3c), three sires were inferred. If seven or eight paternal alleles were present at any loci (Figure 3d), four sires were inferred. For apparent singly sired clutches with strictly paternal or stronger paternal than maternal or embryonic signal strength, we attempted to call paternal genotypes via eggshell and PVM electropherogram comparisons (e.g., Figure 2b vs. 2a). The number of sires of each clutch was subsequently inferred via parentage and sibship analyses using COLONY2 (Wang 2012). Analyses were conducted under the assumptions of polygamy in both sexes with maternal genotypes via eggshells provided as candidate mothers (loggerhead maternal genotypes, Table S3; green turtle maternal genotypes, Table S4). Parentage and sibling inferences were generated under medium runs using strict matching criteria with unknown allele frequencies that were not updated during runs and no sibship size prior.

Since the ability to distinguish sires depends on population allele distributions and allele sharing among parental groups, we quantified microsatellite panel power via estimation of non‐exclusion probabilities for identity, sibling identity, and second parents for the species panels based on maternal genotypes using CERVUS (Kalinowski et al. 2007). Second parent exclusion is important in this context as maternal genotypes were known via eggshells. To explicitly compare performance of PVM detection with the standard 20‐hatchling sampling approach, we performed 100 random subsamples without replacement for clutches represented by > 75 offspring with skewed paternal contributions (those where sires contributed ≤ 11% of sampled offspring) to determine the likelihood of detection assuming only 20 hatchlings had been sampled.

## 3 Results

### 3.1 Genotypes

#### 3.1.1 Loggerhead Turtles

Based on maternal genotypes, the loggerhead panel had a non‐exclusion probability of identity of 4.58 × 10^−27^, non‐exclusion probability of sibling identity of 8.39 × 10^−9^, and a second parent non‐exclusion probability of 4.07 × 10^−11^. Embryos were confidently identified and removed from 20 of 27 freshly laid eggs (Table 1). Embryos were not identified in six additional eggs despite attempting to locate them, and embryo removal was uncertain for two additional eggs (Table 1). At least some sperm alleles successfully amplified in all 27 PVMs genotyped. The majority of PVM genotypes were either strictly paternal (12 of 27, 44%; Figure 2a,b) or had stronger paternal than maternal or embryonic signal strength (10 of 27, 37%; Figure 2c,d) (Table 1). Stronger maternal signal strength was rare (3 out of 27 PVMs, 11%; Figure 2e,f), and at least some paternal alleles were still detectable even in these cases. Among the 20 clutches where the embryonic disc was thought to be removed, one PVM genotype appeared primarily embryonic (Cc101). Excluding the paternal alleles present via embryonic contamination in this PVM, only two of 12 unique sperm alleles were present (Table 1). Among the six eggs where the embryonic disc was not identified and removed, one additional PVM was dominated by apparent embryonic DNA (Cc041). Embryonic swamping was inferred based on the presence of only two alleles at most loci, with one shared with the maternal genotype across all loci examined (Table S5).

**TABLE 1:** Summary of male contributions inferred from sperm trapped in the perivitelline membrane (PVM) and offspring samples for Melbourne Beach, Florida, USA marine turtle nests.

Species	Nest ID	ER	PVM signal	PVM sires	Off sires	S1 Off	S2 Off	S3 Off	S4 Off	S1_PAP	S2_PAP	S3_PAP	S4_PAP	UPAD
Cc	41	N	Embryonic	NA	2	5	2			12/19^a^	0/14^a^			0.36
Cc	101	Y	Embryonic	NA	1	6				2/14				0.14
Cc	137	Y	Admix: Mat	3	3	7	2	1		23/27	8/16^a^	5/8^a^		0.71
Cc	140	Y	Admix: Mat	2	2	60	33			27/27	19/19			1.00
Cc	148	Y	Admix: Mat	1	1	19				12/16				0.80
Cc	31	Y	Admix: Pat	1	1	12				23/23				1.00
Cc	32	N	Admix: Pat	1	2	5	2			25/25^a^	0/16^a^			0.65
Cc	80	N	Admix: Pat	1	1	15				21/21				1.00
Cc	102	N	Admix: Pat	1	1	11				26/26				1.00
Cc	117	U	Admix: Pat	1	1	15				24/24				1.00
Cc	119	Y	Admix: Pat	2	2	77	9			25/25	21/21			1.00
Cc	138	Y	Admix: Pat	1	1	9				24/24				1.00
Cc	139	Y	Admix: Pat	3	3	5	5	1		23/23^a^	21/21^a^	23/23^a^		1.00
Cc	142	Y	Admix: Pat	1	1	57				28/28				1.00
Cc	144	Y	Admix: Pat	2	2	80	1			26/26	2/11^a^			0.76
Cc	27	N	Pat	2	2	18	2			30/30	25/25^a^			1.00
Cc	45	Y	Pat	1	1	9				30/30				1.00
Cc	46	Y	Pat	1	1	17				32/32				1.00
Cc	49	Y	Pat	2	2	6	3			30/30	26/26^a^			1.00
Cc	57	Y	Pat	2	2	5	2			29/29^a^	26/26^a^			1.00
Cc	62	Y	Pat	1	1	9				32/32				1.00
Cc	100	Y	Pat	1	1	14				30/30				1.00
Cc	106	U	Pat	2	2	15	5			30/30	22/22^a^			1.00
Cc	123	Y	Pat	1	1	10				30/30				1.00
Cc	127	Y	Pat	1	1	10				31/31				1.00
Cc	143	Y	Pat	1	1	6				30/30				1.00
Cc	146	Y	Pat	2	2	34	16			32/32	21/21			1.00
Cm	213	Y	Admix: Pat	1	1	122				21/21				1.00
Cm	254	Y	Admix: Pat	2	3	89	11	1		17/17	16/17	0/6^a^		0.83
Cm	208	Y	Pat	3	3	7	6	3		24/24	18/18	10/10^a^		1.00
Cm	214	Y	Pat	2	2	67	38			26/26	21/21			1.00
Cm	218	Y	Pat	3	3	58	15	5		24/24	9/18	8/16^a^		0.71
Cm	219	N	Pat	3	2	10	7			26/26	18/18			1.00
Cm	222	Y	Pat	3	3	60	53	21		25/25	17/19	17/17		0.97
Cm	228	Y	Pat	4	4	82	10	6	3	24/24	17/17	17/17	13/13^a^	1.00
Cm	238	Y	Pat	1	1	111				26/26				1.00
Cm	250	Y	Pat	4	4	39	34	21	18	26/26	22/22	13/13	13/13	1.00
Cm	256	Y	Pat	1	1	109				25/25				1.00
Cm	261	Y	Pat	1	1	16				26/26				1.00
Cm	264	Y	Pat	1	1	94				24/24				1.00

*Note:* Species: Cc, *Caretta caretta*; Cm, *Chelonia mydas*. ER (Embryo Removed): N, no; U, attempted, but uncertain; Y, yes. PVM signal: Pat, paternal, no embryonic or maternal signal detected; Admix: Mat, admixed maternal and paternal but with maternal peaks taller than paternal; Admix: Pat, admixed but with paternal peaks taller than maternal. PVM sires is the minimum number of sires inferred based on sperm alleles in the PVM; NA indicates that no paternal inference was attempted due to domination of embryonic signal. Off sires is the number of sires inferred from analysis of offspring genotypes in COLONY. S(*x*) Off: The number of genotyped offspring attributed to respective sires of each clutch as inferred by COLONY analyses. Paternal alleles present (PAP) is the total number of unique paternal alleles detected in the PVM relative to those inferred from offspring genotypes. For admixed PVMs, this number conservatively excludes shared maternal alleles. Unique paternal alleles detected (UPAD) is the proportion of offspring paternal alleles detected in the PVM genotype.

^a^ Some paternal alleles may be unaccounted for due to five or fewer offspring genotyped.

Each clutch was represented by six to 93 offspring genotypes. Most sampled offspring (620/650, 95.4%) yielded genotypes of sufficient quality for inclusion in COLONY analyses (Table S6). Samples that failed to amplify at six or more loci and had extensive apparent null alleles (> five homozygous loci) were excluded from parentage analyses. Offspring genotypes indicated the presence of at least 41 sires across the 27 clutches examined (Table S7). COLONY analysis yielded high confidence, complete paternal genotypes for most inferred sires (Table S8). High confidence full paternal reconstruction was not possible for secondary and tertiary sires via offspring genotypes when five or fewer offspring were present in the dataset.

#### 3.1.2 Green Turtles

Based on maternal genotypes, the green turtle microsatellite panel had a non‐exclusion probability of identity of 2.93 × 10^−18^, non‐exclusion probability of sibling identity of 1.20 × 10^−6^, and a second parent non‐exclusion probability of 3.00 × 10^−7^. All green turtle PVMs were either paternal or had a stronger paternal than maternal signal, and sperm alleles were detected in all 13 green turtle PVMs genotyped (Table S5). Most sampled offspring (1117/1119, 99.8%) yielded genotypes of sufficient quality for inclusion in COLONY analyses (Table S9). COLONY analyses of offspring and maternal genotypes supported contributions from at least 29 sires across the 13 clutches (Table S10) and yielded high‐confidence paternal genotypes for most males that sired at least five offspring (Table S11).

### 3.2 PVM and Offspring‐Informed Sire Inference Comparisons

#### 3.2.1 Loggerhead Turtles

Offspring genotypes confirmed embryonic swamping of the PVM in two egg samples (Cc041 and Cc101) with only one maternal and one paternal allele present across most loci. Rarely, a second paternal allele was present in a small minority of loci (Table S5). These clutches were excluded from MP inferences. Of the remaining 25 clutches, the minimum number of males inferred based on sperm alleles that amplified in the PVM extract was corroborated by offspring ground‐truthing in 24 nests (Table 1). The exception was clutch Cc032's secondary sire inferred from offspring genotypes but not detected in the PVM alleles. Despite maternal DNA swamping in three nests that resulted in paternal null alleles in PVM genotypes, sufficient sperm allele amplification occurred to infer the correct number of sires for these nests (Table 1). Except for clutch Cc032, PVM sperm allele amplification was sufficient to detect all sires represented by ≤ 11% of sampled offspring (Table S12). When considering only deeply sampled clutches where paternal contribution inferences were more confident, PVM sire detection for these minor contributions was consistent with detection probabilities assuming 20‐hatchling sampling (Table 2).

**TABLE 2:** Detection of sires that contributed ≤ 11% of offspring from clutches with at least 75 offspring sampled.

Clutch	Sire	Contribution	PVM detection	20‐Off detection
Cc119	Sire 2	0.1047	Yes	0.93
Cc144	Sire 2	0.0123	Yes	0.20
Cm218	Sire 3	0.0641	Yes	0.80
Cm228	Sire 2	0.0990	Yes	0.91
Cm228	Sire 3	0.0594	Yes	0.75
Cm228	Sire 4	0.0297	Yes	0.41
Cm254	Sire 2	0.1089	Yes	0.90
Cm254	Sire 3	0.0099	No	0.24

*Note:* Perivitelline membrane (PVM) detection indicates whether the sire was correctly inferred from the PVM genotype. 20‐Off detection was the proportion of sire detections based on 100 random subsamples of 20 offspring from the paternal contribution distribution inferred from parentage analyses.

Except for two clutches (Cc032 and Cc144), all paternal alleles inferred from offspring genotypes were present in the PVMs with paternal or stronger paternal than maternal signal strength (Table 1). This includes cases where secondary or tertiary sires contributed minor proportions of sampled offspring, for example, secondary sire for clutch Cc119 (11%), secondary sire for clutch Cc027 (11%), and the tertiary sire for clutch Cc139 (9%). Across the dataset, we called 36 unique paternal alleles from PVM extracts that were not detected in sampled offspring genotypes (representing 11 sires in eight eggs; Table 3; Table S5). These only occurred when inferred sires were represented by five or fewer offspring. In all cases, the COLONY‐inferred paternal genotype was homozygous at these loci or shared a maternal allele, suggesting that these PVM‐called alleles represented paternal contributions from the inferred sires (Table 2; Tables S5 and S8).

**TABLE 3:** Unique paternal alleles (UPA) called from the PVM extract that were not detected among sampled offspring.

Clutch	UPA	Sire	Off
Cc027	5	Secondary	2
Cc032	1	Primary	5
Cc041	1	Primary	5
Cc049	3	Secondary	3
Cc057	1	Primary	5
Cc057	9	Secondary	2
Cc106	2	Secondary	5
Cc137	1	Tertiary	1
Cc139	1	Secondary	5
Cc139	11	Tertiary	1
Cc144	1	Secondary	1
Cm208	3	Tertiary	3
Cm219	6	Tertiary^a^	NA^a^
Cm228	2	Quarternary	3

*Note:* Sire is the inferred contributor of the allele(s) and the number of sampled offspring (Off) assigned to him via parentage analyses in COLONY.

^a^ The inferred tertiary sire for Cm219 was not detected via offspring genotypes.

#### 3.2.2 Green Turtles

**FIGURE 4:**
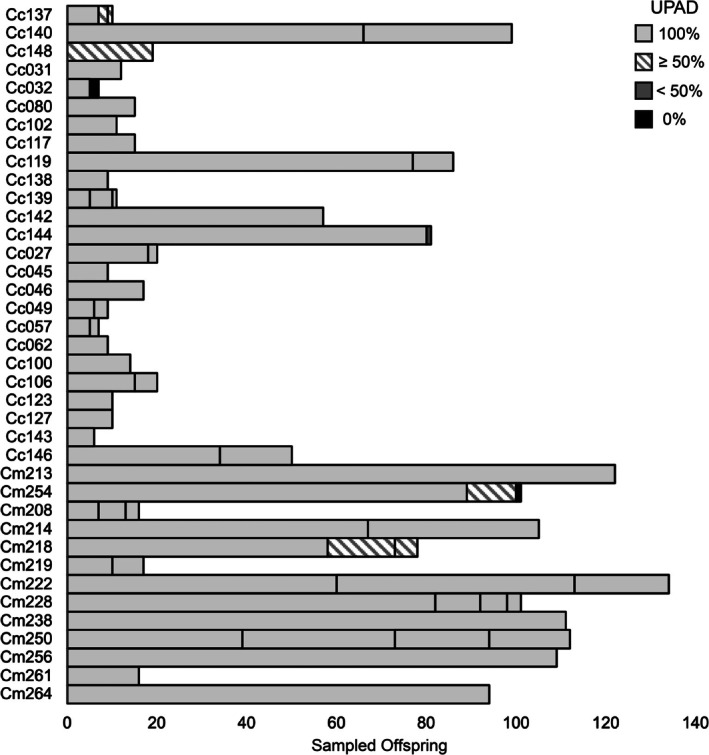
Inferred paternal contributions to loggerhead turtle and green turtle nests based on offspring genotyping. Clutch ordering follows Table 1. Vertical line breaks inside bars indicate different sires. Colors denote the proportion of unique paternal alleles identified in offspring that were detected (UPAD) in the perivitelline membrane (PVM) genotype.

The minimum number of sires inferred from the PVMs was corroborated by offspring genotypes in 11 of 13 clutches sampled. The first exception was Cm254, where a tertiary male sired a single hatchling (< 1% of 101 offspring sampled) that went undetected in the PVM. The second exception was CM219, where a tertiary male inferred from the PVM was not detected among the 17 offspring genotyped (Table 3). This tertiary male was inferred based on the presence of unique paternal alleles at six loci, including a seventh allele at locus Or2, confirming a fourth parent (Tables S4, S5, and S11). Sperm amplification was sufficient to detect five of six sires that contributed ≤ 11% of sampled offspring in their respective clutches (Table S13; Figure 4). Random subsampling of these clutches demonstrated that PVM sire detections were comparable to detection probabilities from sampling 20 hatchlings per clutch (Table 2). The quaternary sire for Cm228 that contributed 3% of offspring was detected via PVM genotyping but was not encountered in 59% of hatchling subsampling trials. The Cm254 tertiary sire that was not identified via PVM genotyping also went undetected in 76% of 20‐hatchling subsampling scenarios (Table 2).

All known paternal alleles inferred from offspring were present in the PVMs for 24 of the 30 inferred sires, including all singly sired clutches and the primary contributor of all multiply sired clutches. Despite some paternal allele dropout, 423 of 455 (93%) of paternal alleles from multiply sires clutches were detected in the PVM. These null alleles were not always correlated with relative contributions inferred from offspring genotypes. For example, all unique paternal alleles were present from clutch Cm222's tertiary sire that contributed 16% of sampled offspring, but the secondary sire that fathered 40% of sampled offspring had two alleles missing from PVM calls (Table 1). Despite the presence of paternal null alleles, sufficient sperm alleles amplified to detect a quaternary male that contributed only three hatchlings in a sample of 101 (3%). All unique paternal alleles present in his three offspring were detected in the PVM (Table 1).

We called 11 unique paternal alleles from PVM extracts that were not detected in sampled offspring genotypes (Table 3; Table S5). Apart from the inferred tertiary sire of Cm219, which was not corroborated by COLONY parentage analyses, these alleles were only present when sires were represented by three or fewer offspring. In all cases, the COLONY‐inferred paternal genotype was homozygous at these loci or shared a maternal allele (Table 2; Tables S5 and S11).

### 3.3 Single‐Sire PVM Genotype Inference Accuracy

#### 3.3.1 Loggerhead Turtles

For the 14 clutches with strictly paternal or stronger paternal than maternal signal and initially inferred as single paternity via PVM genotyping (including Cc032 where the secondary sire alleles were not detected in the PVM), all paternal alleles inferred via offspring genotypes were present in the PVM. Given the strong signal strength disparity between the inferred sires of Cc144 (80 and 1 offspring, respectively), we attempted to call the primary contributor's genotype for inclusion in paternal genotype comparisons. Of the 480 paternal allele calls attempted (15 samples × 16 loci × 2 alleles) via PVM and eggshell comparisons (Table S14), 475 (99%) were corroborated based on offspring genotypes (Table S6). One discrepancy involved allele 133 present in the CC032 PVM at locus 70 but not detected in this sire's five sampled offspring (Tables S8 and S14). It is unclear whether the second discrepancy represents maternal contamination or a shared allele (allele 237 at locus 31 in the Cc127 PVM). The final three discrepancies were allele calling errors and all involved clutch Cc031 PVM, where maternal signal strength approached that of the paternal fraction (nearly 1:1 average peak RFU ratio). Two alleles were originally considered maternal leakage when they represented shared alleles, whereas one allele originally treated as shared was maternal leakage overlaid over a homozygous paternal genotype (Table S6).

#### 3.3.2 Green Turtles

Of the 130 paternal allele calls attempted (5 samples × 13 loci × 2 alleles), all were corroborated by offspring genotypes (Tables S11 and S15). No paternal alleles were missing from the PVM calls of singly sired clutches.

## 4 Discussion

### 4.1 Multiple Paternity Inferences

Previous research in artificially inseminated chickens detected substantial paternal allelic dropout, with only 40% of egg PVM samples yielding the full, expected paternal genotypes (Martínez and Burke 2003). In the present study, all known paternal alleles were recovered from the PVMs for 32 of 41 (78%) loggerhead turtle sires and 24 of 29 (83%) green turtle sires. Excluding the PVMs with obvious embryonic and maternal swamping, these full PVM paternal genotype detection rates were substantially improved for loggerheads (30 of 32, 94%). These metrics included several males that contributed ≤ 11% of sampled offspring. For all males that contributed > 11% of sampled offspring, we recovered 98% of unique paternal alleles from the PVM genotypes from each species. Despite the presence of some paternal allele dropout in the PVM extracts, there was sufficient sperm amplification to correctly identify all sires that contributed at least 1% of sampled offspring with one exception. Overall, these results are promising and suggest that PVM sampling yields MP inferences comparable to the current standard hatchling sampling approach with substantially less effort and cost (e.g., genotyping only a single PVM sample per clutch rather than 20 offspring).

The paternal allelic dropout previously documented in the chicken artificial insemination study was attributed to a combination of low DNA concentrations as well as preferential amplification of shorter sperm alleles (Martínez and Burke 2003). These investigators recommended choosing dinucleotide loci with short fragment lengths and small array ranges with no more than 10 base pairs across alleles. All loggerhead turtle markers used in the current study were tetranucleotide loci with array ranges spanning 40 to 75 base pairs and with fragment lengths up to 400 base pairs. Despite this, null alleles were primarily limited to PVMs that were swamped by embryonic or maternal DNA. There was no evidence of preferential amplification causing the paternal null alleles in the PVMs. For example, the PVM paternal null alleles from Cc032's inferred secondary sire were often shorter than those detected for the primary sire (e.g., locus 31 null allele 225, alleles 229 and 241 from primary sire present; locus 74 null allele 241, alleles 257 and 261 from primary sire present; Table S5). Differences in relative DNA concentration are the more likely explanation for the PVM null alleles. In all cases except for Cc032, loggerhead turtle sires contributing at least 1% of sampled offspring and in PVMs where paternal signal strength was stronger than maternal had no apparent allele dropout. It's unclear why Cc032 was the exception. One possibility is that the secondary sire's offspring may have been over‐represented among the dead in nest while they could have represented < 10% of the overall clutch composition. Additional research is warranted to better characterize detection limits, as only eight sires had proportional contributions ≤ 11% in the present study. Nonetheless, seven of these were successfully inferred via the PVM. This ability to detect these minor paternal contributions was facilitated by the increased sampling depth rather than the conventional ~20 hatchlings per clutch sampling. Further, the PVM genotypes yielded 47 alleles not detected among sampled offspring. Although we cannot definitively rule out PCR artifacts, 41 of these were present in corroborated sires at loci inferred as homozygous via parentage analyses, and all these sires were represented by five or fewer offspring.

Green turtle paternal allele inferences were generally as robust as loggerheads but with slightly higher allelic dropout rates in MP clutches. Six of the 13 microsatellite markers used in green turtle analyses were dinucleotide loci (Table S2). Across the green turtle dataset, nine paternal alleles inferred from offspring genotypes were not initially called in the PVM electropherograms because they were interpreted as stutter peaks of primary contributor alleles. These alleles were subsequently confirmed as present but obscured by these stutter peaks. Marker choice is important for maximizing the ability to distinguish unique alleles, so we recommend avoiding dinucleotide loci with strong stuttering patterns. Contrary to previous advice with avian perivitelline layer analyses, we recommend choosing tetranucleotide loci with wide allelic ranges for ease of scoring and to minimize the potential of missed allele calls. As with loggerheads, there was no evidence of preferential amplification causing allele dropout. For example, allele 184 from the tertiary sire of Cm254 was absent in the PVM, but longer alleles 198 and 204 from the primary sire were detected (Table S5).

In the present study, we shallowly sampled a larger number of loggerhead clutches and deeply sampled a smaller number of green turtle clutches. Inferences of technique performance from the smaller number of green turtle clutches should be treated with caution. Nonetheless, sperm allele amplification and allele sharing patterns among parental groups permitted identification of seven of eight sires that contributed ≤ 11% of offspring in deeply sampled clutches in both species. While minimum sire inferences from PVM genotypes were corroborated for both species, the higher frequency of null alleles in green turtles warrants further investigation. Given the relatively small number of skewed parental contributions in the present study, future work should attempt to improve quantification of detection limits of this approach.

### 4.2 Paternal Genotypes in Singly Sired Clutches

In PVMs with strictly paternal or stronger paternal than maternal or embryonic signals, it was possible to call high quality, complete paternal genotypes. Most uncertainty in paternal allele calls was due to the presence of maternal contamination at loci with fewer than four alleles present. Distinguishing between maternal contamination and parental allele sharing was especially difficult as maternal signal strength approached that of paternal. Despite the presence of some maternal contamination, paternal genotype calls from the PVM were highly concordant with those from offspring genotypes for both loggerheads and green turtles.

Where it is possible to directly infer paternal genotypes from the PVM, these genotypes may circumvent the need to genotype live hatchlings for paternal genotype construction. Furthermore, they could unlock additional information beyond breeding sex ratio inferences. Genetic tagging of individual males, through a combination of PVM and offspring genotyping, could inform male breeding frequency. In the longer term, individual genetic tagging could also inform male reproductive longevity in the same way that combined physical and genetic tagging have informed female reproductive lifespans (Shamblin, Ondich, et al. 2021). These may be particularly powerful approaches in relatively small populations where recapture rates are sufficient to support male reproductive periodicity and survival analyses.

Male genotypes could prove useful for population assignment or in characterizing the scale and magnitude of gene flow where population assignments are not feasible. Northwest Atlantic loggerhead turtles have been subdivided into several demographically discrete populations based on female natal homing, known as management units, using maternally inherited mitochondrial DNA markers (Bowen et al. 2005; Shamblin, Bjorndal, et al. 2012). However, nuclear DNA structure is weak or non‐existent among the mitochondrially defined populations, and migration‐mediated or male‐mediated gene flow have been invoked as possible homogenizing factors with respect to nuclear genetic structure (Bowen et al. 2005). If regional gene flow is occurring via these mechanisms, a more holistic picture of genetic connectivity is needed to better resolve breeding sex ratios beyond focal beach estimates. Stable isotope analyses have indicated the presence of adult females representing the central eastern Florida and southeastern Florida management units at MidAtlantic Bight and South Atlantic Bight foraging sites (Ceriani et al. 2023), providing a potential avenue for migration‐mediated gene flow. Such regional scale gene flow could prove invaluable if southern populations are male‐limited. In species and at regional scales where sufficient nuclear genetic structuring exists among nesting populations, for example, Mediterranean loggerheads (Clusa et al. 2018), Atlantic leatherbacks (Dutton et al. 2013), and Pacific green turtles (Roden et al. 2023), PVM genotyping could be useful for elucidating male‐mediated or migration‐mediated gene flow.

### 4.3 Further Refinements

PVM genotyping is a robust technique for capturing breeding male demographic and genetic information despite the presence of maternal contamination and paternal null alleles. Nonetheless, it may be possible to further optimize application of this methodology by addressing a few remaining key questions:

#### 4.3.1 Is Variation in Maternal DNA Signal in PVM Extracts Driven by Intrinsic or Extrinsic Factors?

There was substantial variation in the magnitude of maternal contamination of the PVM extracts across clutches. Relatively stronger maternal signal strength in the PVM led to paternal allele dropout in two of three loggerhead clutches in this study. The presence of maternal contamination also introduced uncertainty of paternal allele calls in singly sired clutches where less than four alleles were present at a particular locus. Eliminating or reducing this maternal signal in the PVM would clarify MP inferences and simplify paternal allele calling from PVM genotypes. Because each female was represented by a single egg in this study, it is unclear whether this variation was influenced by intrinsic or extrinsic factors. If maternal contamination of the PVM is related to sample storage, the PVM isolation process, or DNA extractions, it may be possible to further reduce this maternal signal through protocol modifications. Future research should assess variation in the maternal signal of PVMs across successive clutches of individual females to address the possibility of intrinsic differences among females. If intra‐female variation in maternal contamination occurs across nests, it would suggest that extrinsic factors are driving the contamination. Under this scenario, exploring protocol variations that could maximize paternal signal while minimizing maternal signal could further enhance the efficiency of the PVM genotyping approach.

#### 4.3.2 Do PVM Paternal Signals Vary Across Successive Clutches for Individual Females?

In marine turtles, mating is assumed to occur prior to the initiation of the nesting season such that sperm from all sires should be present when oviposition begins (Hamann et al. 2003). The degree to which sperm may be mixed or stratified has not been well characterized in marine turtles. An MP study of loggerhead turtles nesting in southwestern Florida detected novel sires in later season clutches, invoking the possibility of sampling error, stratification of sperm in the storage tubules, depletion of some paternal contributions over time, mating during the nesting season, or other sperm competition mechanisms that affected fertilization efficiency (Lasala et al. 2020). Species with large sperm storage organs and random sperm mixing are expected to conform to the fair raffle hypothesis of sperm competition (Parker 1990). Stratification of sperm within tubules can lead to deviations from this fair raffle (Parker and Pizzari 2010). The relatively higher frequency of null alleles in green turtles and the apparent lack of correlation between null alleles and paternal contributions suggest that green turtle sperm may be more stratified or clumped than that of loggerhead turtles. Sire inferences from PVMs with well mixed sperm would offer a more complete picture of breeding male availability without any constraints on the sampling period each nesting season. Alternatively, if male contributions prove highly variable across the nesting season, a temporally stratified sampling design might be necessary to fully capture male contributions. Assessing the consistency of sire contributions across successive clutches of individual females should be prioritized as a research objective.

#### 4.3.3 How Do Storage Time and Conditions Influence Sperm Amplification Success in PVM Extracts?

All samples in this study were refrigerated for less than a week prior to PVM isolation. Refrigeration for longer periods prior to PVM isolation would relax logistical constraints, but it's unclear how this might influence genotyping success. In some cases, freezing might be a better option, but it's unknown how this might affect the consistency of egg components and the ability to locate and remove embryos. Refrigeration and freezing may not be feasible at remote field sites, so other storage options such as PVM preservation in saturated salt or ethanol should be explored.

### 4.4 A Path Towards Indices of Male Availability

Male biology and reproductive contributions remain among the least understood aspects of marine turtle ecology, representing a critical gap in our ability to assess population viability and inform conservation strategies (Fuentes et al. 2025; Wallace et al. 2025). Reduced male production has been identified as a key vulnerability under climate change (Patrício et al. 2021), and recent evidence suggests long‐term declines in male hatchling output in several important rookeries (Jensen et al. 2018; Meylan et al. 2024; Ceriani and Casale 2025).

Our results demonstrate that genotyping sperm trapped in the PVM provides a reliable and efficient alternative to hatchling‐based sampling for inferring breeding male contributions in marine turtles. Compared with the standard approach of genotyping ~20 hatchlings per clutch, PVM genotyping requires only a single egg per clutch yet appears to achieve comparable power in detecting MP and reconstructing paternal genotypes for single paternity clutches. In addition to reducing cost and effort, the use of a fresh egg functions as a voucher sample: it substantially increases the likelihood that the intended sample size is obtained, whereas hatchling‐based designs are often compromised when nests fail, are lost due to predation or erosion, or when emergence events are missed. Nonetheless, given the low levels of embryonic and maternal swamping documented in this study, collection of additional samples (~10% more than target sample sizes) would provide a buffer in cases where swamping occurs and precludes downstream analyses. This scalability, reliability, and reduced cost are critical advantages for implementing rigorous, long‐term, multi‐site monitoring of male availability in marine turtle populations.

Breeding male inferences from PVM sperm allele genotypes could provide multiple metrics that may serve as indices of breeding male availability across space and through time: (1) the proportion of MP at focal beaches, (2) the average number of males per clutch at focal beaches, and (3) the proportion of polygyny detections. Taken together, these metrics demonstrate the potential of PVM genotyping as a scalable tool for investigating male reproductive ecology in marine turtles. Baseline sampling would be necessary to establish typical annual variation across successive breeding seasons. Following establishment of a baseline, sampling could be conducted continuously or revisited at regular temporal intervals.

### 4.5 Implementation Considerations

Power to detect MP and estimate BSR are affected by the genetic markers employed (number of loci, allelic diversity, and allele sharing patterns among parents) and mating system parameters (the number of sires, paternal contribution skewness within clutches, and degree of polygyny) (Quennessen et al. 2025). Maximizing the ability to detect MP may require nearly full clutch sampling for absolute detection as the number of sires and contribution skewness increase, whereas simulations have confirmed that sampling fewer offspring from more clutches increases confidence in BSR inferences (Quennessen et al. 2025). Study design should therefore be tailored to address specific research goals. Pilot studies are warranted for new populations and species to properly contextualize MP and BSR inferences prior to widescale application.

**FIGURE A1:**
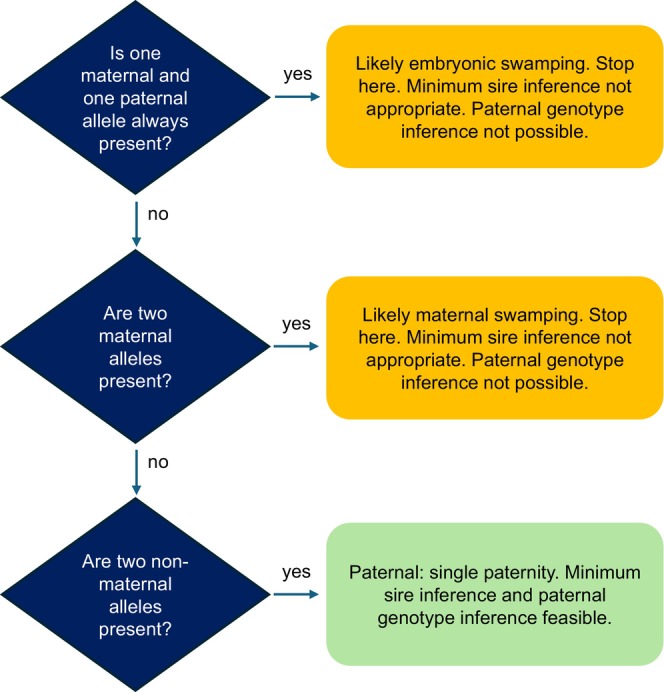
PVM primary signal characterization decision tree when two alleles are present. If more than two alleles are present at most loci, see Figure A2.

**FIGURE A2:**
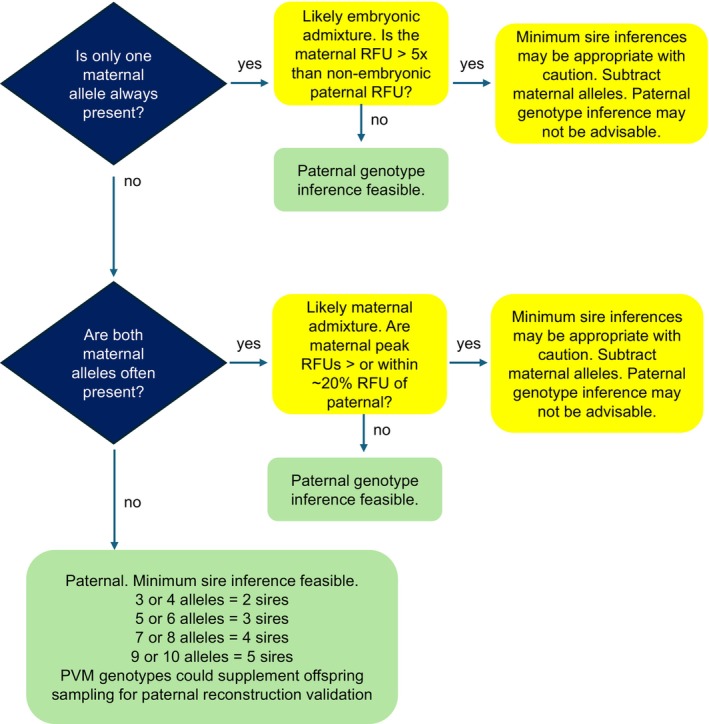
PVM primary signal characterization decision tree, three or more alleles.

A fundamental but sometimes challenging aspect of PVM genotype interpretation is determining whether the signal is primarily paternal or admixed with maternal or embryonic DNA. Making this determination is important because in cases of admixture, maternal alleles should be excluded for minimum sire inferences, and the presence of maternal DNA can impact the interpretation of single paternity genotypes. Distinguishing paternal from admixed signals often requires comparisons with respective maternal genotypes across several loci. When PVM genotypes consist primarily of two alleles across several loci, it's important to determine if these represent paternal genotypes or embryonic or maternal swamping of paternal DNA (Figure A1). When PVM genotypes consist of three or more alleles across multiple loci, these might represent multiple paternity, a mixture of paternal and maternal DNA, or a mixture of paternal and embryonic DNA (Figure A2). The ability and interest in gleaning information from these cases of maternal or embryonic admixture will vary with project goals and investigator risk tolerance. Our study suggests that minimum sire inferences are robust with moderate levels of maternal contamination. However, we recommend conservatively eliminating maternal allele calls from admixed PVM genotypes.

Paternal genotype calling in single paternity clutches was robust to the presence of low to moderate levels of maternal contamination. However, caution is warranted with paternal genotype inferences when maternal peak intensity is within ~25% of or exceeds sperm allele RFUs. Although it is feasible to conservatively estimate the number of sires contributing to each clutch via PVM genotyping, it is typically not possible to assign alleles to specific males in multiply sired clutches in most cases. If reconstructing paternal genotypes is a goal, offspring (live or dead in nest) sampling will be necessary. The offspring sampling effort necessary to achieve this would depend on relative male contributions and study goals. At least six offspring per sire are required to achieve a high probability (> 0.96) of capturing both paternal alleles and to have reasonable confidence in any inferred homozygous genotypes. However, full reconstruction of paternal genotypes is not necessary for confident inference of male identity. High confidence genotypes at a portion of loci via offspring genotyping would suffice for estimating the minimum number of sires contributing to a pool of offspring.

Real‐time PVM genotyping while clutches are incubating could identify multiply sired clutches to facilitate targeted offspring sampling. This would reduce effort and costs associated with generation of paternal genotypes from singly sired clutches where that genotype can be inferred directly from the PVM. A stratified sampling design composed of focal beach PVM characterization supplemented with opportunistic salvaging of dead offspring during nest evaluations at additional sites would facilitate estimation of breeding male indices as well as provide opportunities to characterize male breeding connectivity across sites.

In practical terms, PVM genotyping offers three key advantages over the traditional ~20‐hatchling approach. First, collecting a single fresh egg at or near oviposition avoids investing effort in nests that subsequently fail or are lost before hatchling emergence. Second, PVM genotyping requires only a single sample for minimum sire inferences. Third, although destructive to a single egg, this approach is non‐invasive to hatchlings and nesting females. Together, these attributes establish PVM genotyping as a logistically and financially efficient alternative for inferring male contributions and breeding sex ratios in marine turtles.

## Author Contributions

**Brian M. Shamblin:** conceptualization (equal), data curation (equal), formal analysis (equal), methodology (equal), software (lead), supervision (equal), visualization (lead), writing – original draft (equal), writing – review and editing (equal). **Cheryl L. Sanchez:** conceptualization (equal), data curation (equal), investigation (equal), methodology (equal), supervision (equal), visualization (supporting), writing – original draft (equal), writing – review and editing (equal). **Sean M. Perry:** conceptualization (equal), writing – original draft (supporting), writing – review and editing (supporting). **Simona A. Ceriani:** conceptualization (equal), data curation (equal), funding acquisition (lead), methodology (equal), supervision (equal), visualization (supporting), writing – original draft (equal), writing – review and editing (equal).

## Funding

This work was supported by the Marine Resources Conservation Trust Fund.

## Conflicts of Interest

The authors declare no conflicts of interest.

## Supporting information


**Data S1:** ece373115‐sup‐0001‐TablesS1‐S15.xlsx.

## Data Availability

All primer and multiplex metadata, microsatellite genotypes, and peak relative fluorescence unit metadata are available as Supporting Information.
